# Clinicopathological study on thyroid follicular carcinoma-like renal tumor related to serious hypertension: Case report and review of the literature

**DOI:** 10.1097/MD.0000000000006419

**Published:** 2017-03-24

**Authors:** Hui Wang, Jianpeng Yu, Zhonghua Xu, Gang Li

**Affiliations:** a Department of Urology, Qilu Hospital of Shandong University, Jinan; b Department of Urology, Dezhou People Hospital, Dezhou City, Shandong Province; c Department of Urology, Second Hospital of Tianjin Medical University, Tianjin Institute of Urology, He Xi District, Tianjin, China.

**Keywords:** hypertension, kidney, thyroid follicular-like renal tumor

## Abstract

**Rationale:**

Thyroid carcinoma-like tumor of the kidney (TLFCK) is an extremely rare variant of renal cell carcinoma. Most cases were incidentally found, while we report the first case of TLFCK presented with hypertension.

**Patient concerns:**

A 25-year-old woman was admitted to our hospital presenting with hypertension for ∼20 months, without gross hematuria, weight loss, and flank pain.

**Diagnoses:**

Imaging studies revealed a right renal mass with multiple calcifications. Histologically, the tumor had striking follicles with dense, colloid-like material resembling thyroid follicular carcinoma while the tumor cells were negative for thyroid markers (thyroglobulin and thyroid transcription factor-1).

**Interventions:**

The patient successfully underwent nephron sparing surgery with an uneventful recovery.

**Outcomes:**

Hypertension returned to normal without any medication interference. Two years after surgery, the patient is still in good health without recurring disease or related hypertension.

**Lessons:**

Recognition of the cytomorphological features of TLFCK can avoid misdiagnosis of this renal tumor as a metastatic carcinoma and the objective of surgical management is to remove the tumor and preserve renal function.

## 1 Introduction

Thyroid carcinoma-like tumor of the kidney (TLFCK) is a new entity and was described firstly in 1996 by Angell et al.^[1]^ Nevertheless, the nomenclature of TLFCK cannot be found in the World Health Organization Classification of renal carcinoma in 2004. Morphologically, TLFCK resembles follicular carcinoma of the thyroid gland containing abundant colloid-like material, rather than a metastatic thyroid neoplasm. Its clinical evolution and prognosis remain unclear. Currently, only several cases have been reported in the English literature.^[2–4]^ Here, we describe a unique case with hypertension and a review of the literature.

## 2 Case report

**Figure 1:**
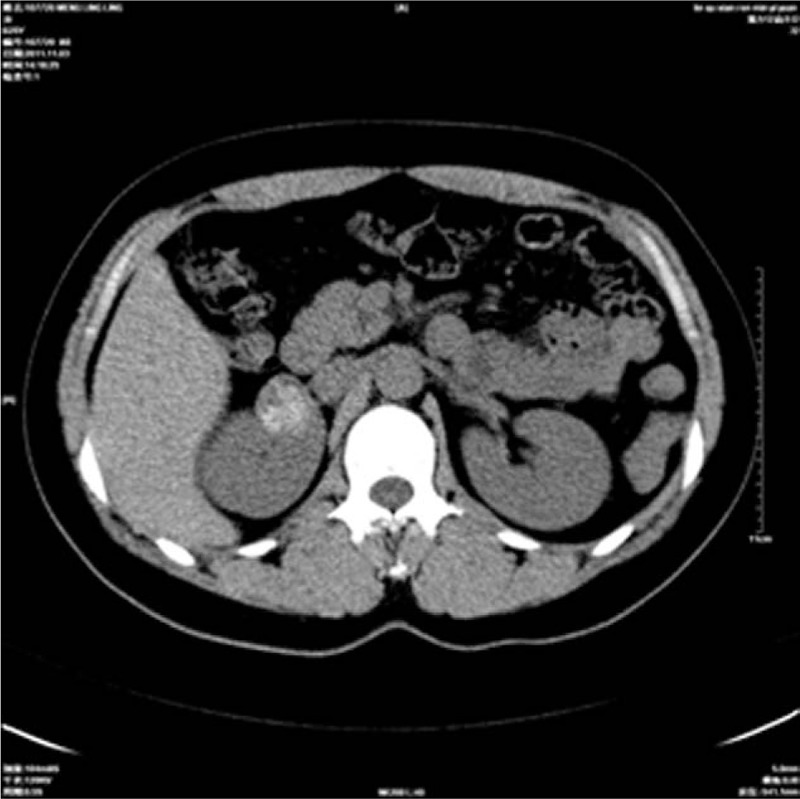
Non-contrast CT demonstrated a heterogeneous mass with obvious calcification and well-defined border measuring about 30 mm in the right kidney. CT = computer tomography.

**Figure 2:**
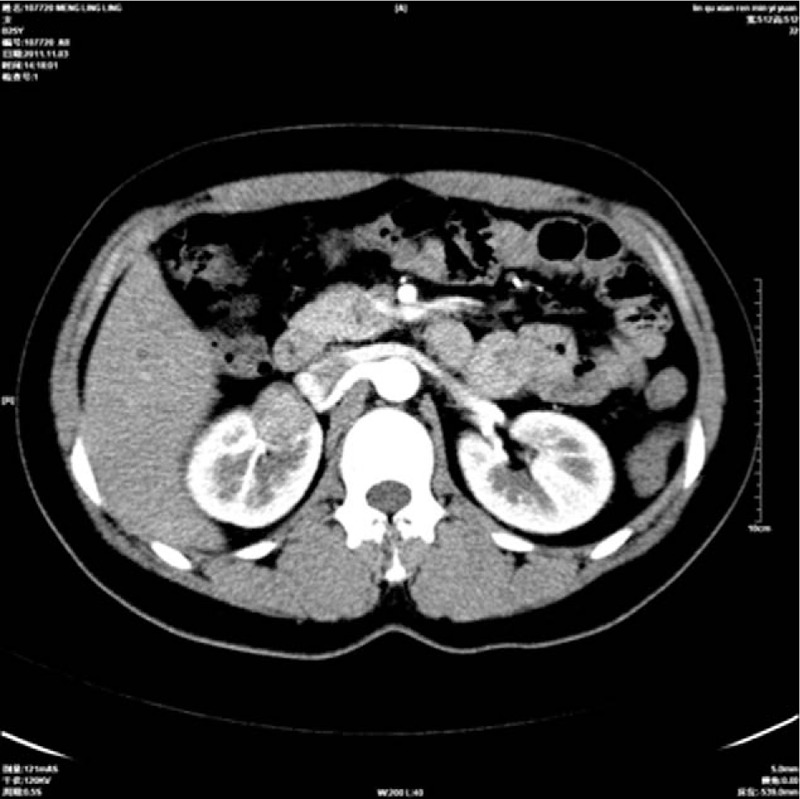
Post-contrast CT revealed the mass was with moderately inhomogeneous enhancement. CT = computer tomography.

A 25-year-old woman was admitted to our hospital on 28 February 2011 presenting with hypertension for ∼20 months. She had no gross hematuria, weight loss, and no flank pain. Physical examination revealed the blood pressure was 200/130 mm Hg (1 mm Hg = 0.133 kPa). No palpable lesion was found in the thyroid gland and abdominal examination showed no abnormalities. There was no history of hypertension in her family and her past medical history is not contributory. Results of routine laboratory tests, including blood regular test, urine analysis, and biochemical investigations of renal, liver, and thyroid function parameters, were all within normal limits. Urinalysis was normal. Radiographically, chest X-ray was negative and abdominal ultrasound showed the presence of an echo-inhomogeneous mass in right kidney. Non-contrast computer tomography (CT) demonstrated a heterogeneous and well-circumscribed soft tissue mass measuring about 30 mm adjacent to the right renal hilum, and there was no evidence of capsule penetration or infiltrative growth pattern. Most renal parenchyma structures were normal and obvious calcification was detected in the lesion (Fig. 1). No enlarged lymph nodes were found in retroperitoneal space. Post-contrast CT revealed moderately inhomogeneous enhancement after contrast (Fig. 2). All this combined with radiological finding and clinical presentation led to the diagnosis of reninoma and the patient underwent surgical exploration. During surgery, a 3-cm mass extended to the right renal hilum. The patient successfully underwent nephron sparing surgery with an uneventful recovery. Hypertension returned to normal without any medication interference. Two years after surgery, the patient is still in good health without recurring disease or related hypertension.

## 3 Pathological results

**Figure 3:**
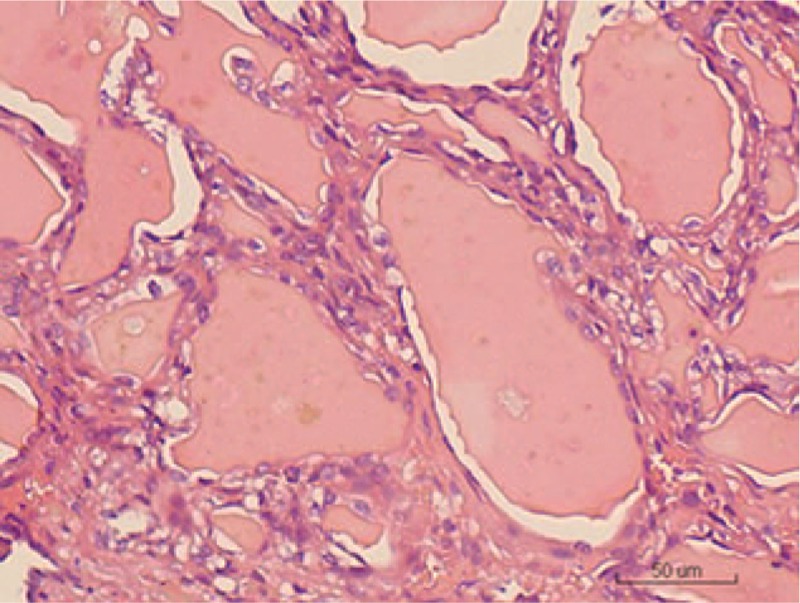
Histologic features of thyroid follicular-like renal carcinoma: follicular architecture of the neoplasm composed of macro- and microfollicles filled with colloid-like material (hematoxylin and eosin, original magnification ×200).

**Figure 4:**
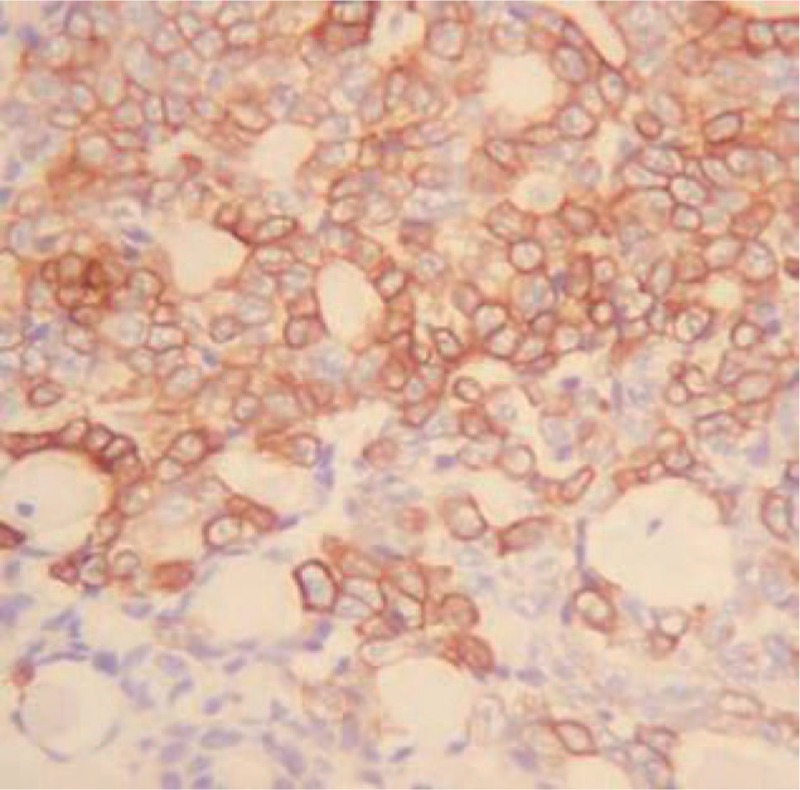
Immunophenotype of thyroid-like follicular carcinoma; a diffuse immunoreactivity for EMA (original magnification × 200). EMA = epithelial membrane antigens.

Grossly, the specimen revealed a white-yellow area on cross-section with multiple cystic areas containing a jelly material and an intact capsule. The cut surface of the specimen was reddish and brownish in color. Histologically, the most striking feature was the presence of TLFCK-like component, the tumor was made up of flat cells forming one layered micro-, normo-, and macrofollicular structures containing abundant eosinophilic colloidal material. The tumor was composed of a striking follicular architecture and microfollicles filled with eosinophilic colloid material that was like a thyroid follicular tumor (Fig. 3). Immunohistochemical analysis was performed and the tumor cells were found to be focally and moderately positive for expressed epithelial membrane antigens (Fig. 4), vimtin, cytokeratins (CK) 20, and CK7. Nevertheless, the cells were negative for cluster of differentiation (CD) 117, thyroid transcription factor-1 (TTF-1), thyroglobulin (TG), chromogranin A, and synaptophysin. Moreover, no radiological abnormalities were found in the thyroid, mediastinum, or pelvis.

## 4 Discussion

TLFCK is an extremely rare renal carcinoma that morphologically mimics follicular carcinoma of the thyroid. Additionally, it has not been identified as any known subtypes of renal cell carcinoma in the 2004 WHO classification.^[5]^ Sporadic cases have been reported with emphasis on histopathologic features in the literature.^[6,7]^ However, clinical features especially associated with hypertension have not yet been documented in the literature to date.

The clinical behavior of TLFCK is unpredictable; the explanation for TLFCK tumor-associated hypertension remains unclear. In our case, hypertension regresses spontaneously after a curative surgery that has not been previously reported. It has been postulated that hypertension was related to rennin which TLFCK may have secreted. Although the tumor can be identified by using various radiological imaging modalities such as US, CT scan, and MRI, these techniques cannot definitely be categorized as determinant diagnoses just based on their radiological characteristics. Presently, CT is the preferred imaging method for establishing and characterizing of a renal tumor. CT provides a valuable complement to existing diagnostic imaging; the appearances are usually nonspecific, the image can only suggest the tumor-like lesions, and the differential diagnosis from renal cell carcinoma have not been established. The abundance of calcification is an unreliable and a variable finding in renal malignant tumors. Awareness of the radiological features of this disease is therefore critical. In our case, the lesion was characterized by abundant calcification, which in the evaluation of TLFCK is still requested for further study. The renal metastasis has similar appearances to the primary malignant lesions found in the CT scan. The final diagnosis usually depends on the clinical history and the pathologic condition and not on the CT and radiological appearances.

The clinical presentation and radiological character are nonspecific; in our case, tumor-associated hypertension is similar to the juxtaglomerular cell tumor of the kidney which was a rennin-secreting tumor.^[8]^ TLFCK was a special tumor that can be verified by histological characteristics. Its morphological features were characterized by a striking follicular architecture composed of macro- and microfollicles filled with amorphous eosinophilic colloid-like material that mirrored follicular carcinoma of the thyroid. The presumptive existence of a new subset of nephroblastoma displayed papillary thyroid carcinoma-like histology along with TTF-1 and TG immunoexpressions. TG and TTF-1 are reliable markers for thyroid follicular carcinoma and the final pathological diagnosis should be straightforward in the event of TTF-1 and TG immunopositivity. Nevertheless, a papillary thyroid carcinoma-like tumor of the kidney should be taken into consideration for differential diagnosis.^[9]^ The major differential diagnoses of TLFCK included metastatic thyroid tumor, papillary renal-cell carcinoma associated with a macrofollicular pattern resembling TLFCK, and carcinoid tumor.^[10]^ Distinguishing primary TLFCK from thyroid cancer metastatic to the kidney cannot be problematic. The striking follicular architecture featured prompts differential diagnosis. Renal metastasis of thyroid follicular carcinoma is a rare event, with only few cases described.^[11]^ Moreover, metastatic thyroid tumors usually preserve a thyroid-specific immunoreaction profile for TG and TTF-1, whereas a secondary renal tumor has to be shown immunonegative for thyroid markers before being considered as a true primary. Histology becomes mandatory for a definitive diagnosis.

When assuming a primary TLFCK of the kidney, primary thyroid carcinoma especially ectopic thyroid tumor must be expelled, although this malignant transformation is extremely rare. In our case, clinical and radiological investigations were negative for both orthotopic and heterotopic thyroid sites. So the final pathological diagnosis was primary thyroid-like follicular carcinoma of the kidney. Postoperative follow-up of 2 years, the patient is alive without relapse or metastases signs. To be surprising, the hypertension was cured without any other medical interference. So the presence of hypertension was sure to have an association with the tumor. The tumor can be successfully managed with the correct surgical management. A malignant clinical course necessitates a long-term follow up, whereas in our case the prognosis is good.

## 5 Conclusions

TLFCK is a rare neoplasm of the kidney that should be distinguished from its malignant mimickers, such as metastatic carcinoma from a thyroid primary and less importantly from a carcinoid tumor. Recognition of the cytomorphological features of TLFCK can avoid misdiagnosis of this renal tumor as a metastatic carcinoma. Once the diagnosis has been established, the objective of surgical management is to remove the tumor and preserve renal function; all therapeutic regimens are more standardized in improving outcome. Although the overall prognosis is favorable, recurrence and metastases of TLFCK may occur.
